# Operative Outcome and Patient Satisfaction in Early and Delayed Laparoscopic Cholecystectomy for Acute Cholecystitis

**DOI:** 10.1155/2014/162643

**Published:** 2014-08-14

**Authors:** Aly Saber, Emad N. Hokkam

**Affiliations:** ^1^Department of General Surgery, Port-Fouad General Hospital, 19 Al-Guish Street, Port Fouad, Port Said, Egypt; ^2^Department of Surgery, Faculty of Medicine, Suez Canal University, Ismailia, Egypt

## Abstract

*Introduction*. Early laparoscopic cholecystectomy is usually associated with reduced hospital stay, sick leave, and health care expenditures. Early diagnosis and treatment of acute cholecystitis reduce both mortality and morbidity and the accurate diagnosis requires specific diagnostic criteria of clinical data and imaging studies. *Objectives*. To compare early versus delayed laparoscopic cholecystectomy regarding the operative outcome and patient satisfaction. *Patients and Methods*. Patients with acute cholecystitis were divided into two groups, early (A) and delayed (B) cholecystectomy. Diagnosis of acute cholecystitis was confirmed by clinical examination, laboratory data, and ultrasound study. The primary end point was operative and postoperative outcome and the secondary was patient's satisfaction. *Results*. The number of readmissions in delayed treatment group B was three times in 10% of patients, twice in 23.3%, and once in 66.7% while the number of readmissions was once only in patients in group A and the mean total hospital stays were higher in group B than in group A. The overall patient's satisfaction was 92.66 ± 6.8 in group A compared with 75.34 ± 12.85 in group B. *Conclusion*. Early laparoscopic cholecystectomy resulted in significant reduction in length of hospital stay and accepted rate of operative complications and conversion rates when compared with delayed techniques.

## 1. Introduction

Early cholecystectomy is the optimal treatment for acute cholecystitis using established optimal surgical treatment for each grade of severity. Several studies have shown that early laparoscopic cholecystectomy conducted within 72–96 hours after the onset of symptoms is usually associated with advantages such as reduced hospital stay, sick leave, and health care expenditures and no disadvantages with regard to mortality and morbidity [1]. Early diagnosis and treatment of patients with acute cholecystitis reduce both mortality and morbidity and the accurate diagnosis requires specific diagnostic criteria of clinical data and imaging studies [2]. The typical ultrasound image of acute cholecystitis demonstrates gallbladder swelling, wall thickening with sonolucent layers, massive debris, and the stone impaction in the cystic duct [3].

## 2. Objectives

The aim of this study was to compare early versus delayed laparoscopic cholecystectomy regarding the operative outcome and patient satisfaction.

## 3. Patients and Methods

A total of 120 patients with acute cholecystitis were enrolled to this prospective randomized study from April 2009 to November 2014 at Port-Fouad General Hospital and Suez Canal University Hospital. Patients were divided according to the timing of surgical technique into two main groups, early (A) and delayed (B). Delayed surgical interference was done after 6 weeks to 8 weeks from the onset of symptoms to allow resolution of the acute inflammation of the gallbladder* while early laparoscopic cholecystectomy was done within 72 hours* [4]. Written consent was obtained from all patients or first degree relatives before the management procedure and the local ethics committee approved the study.

### 3.1. Preoperative Workup

The diagnosis of acute cholecystitis was confirmed by clinical examination, laboratory data, and ultrasound study. Ultrasonography findings were confirmed when sonographic Murphy sign with tenderness on ultrasound probing was elicited, thickened gallbladder wall >4 mm and enlarged gallbladder with long axis diameter >8 cm, short axis diameter >4 cm, sonolucent layer in the gallbladder wall, striated intramural lucencies, and pericholecystic fluid collection [2].

### 3.2. Grading of Acute Cholecystitis

Grade I: mild acute cholecystitis is defined as acute cholecystitis in a healthy patient with only mild inflammatory changes in the gallbladder. Grade II: moderate acute cholecystitis is diagnosed when palpable tender mass is in the right upper abdominal quadrant with marked local inflammation in the US together with WBC count >18 000/mm^3^. Grade III: severe acute cholecystitis is accompanied by organ dysfunctions.

### 3.3. Operative Technique

Laparoscopic cholecystectomy was performed by the treating surgical team consisting of a consultant surgeon and an assistant professor of surgery using the standard 4-trocar technique. Gallbladder contents were aspirated in cases with gallbladder distension. Meticulous dissection was paid to identify the structures in Calot's triangle and attempts of retrograde dissection of the gallbladder starting at the fundus were done in case of severe inflammation and anatomical difficulty of the pericystic space. We used plastic bags for gallbladder removal from the abdomen for prevention of wound infection and falling of stones.

### 3.4. Randomization

Randomization was performed prior to study commencement as follows: opaque envelopes were numbered sequentially from 1 to 120. A computer-generated table of random numbers was used for group assignment; if the last digit of the random number was from 0 to 4, assignment was to group A (early laparoscopic cholecystectomy group (EL)), and if the last digit was from 5 to 9, assignment was to group B (delayed laparoscopic cholecystectomy group (DL)). The assignments were then placed into the opaque envelopes and the envelopes were sealed. As eligible participants were entered into the study, these envelopes were opened in sequential order to give each patient his or her random group assignment. The envelopes were opened by the operating surgeon after patient consent indicating the agreement of the study protocol and just prior to the surgery.

### 3.5. End Points

The primary end point of the study was operative and postoperative outcome and the secondary end point was patient's satisfaction. Patient's satisfaction was measured according to the recurrent attacks of pain, times of readmissions, length of hospital stay, and morbidity related to surgery.

### 3.6. Statistical Analysis

Data collected were processed using SPSS version 15 (SPSS Inc., Chicago, IL, USA). Quantitative data were expressed as means ± SD while qualitative data were expressed as numbers and percentages [%].

## 4. Results

A total of 120 cholecystectomies were performed, 60 for both early and delayed laparoscopic cholecystectomies. Concerning the demographic data, there was no statistically significant difference between the two groups regarding age, sex, and body mass index. Grades and severity of acute cholecystitis were traced in both groups according to the clinical finding, laboratory data, and imaging studies. Only grade I and grade II were included as shown in Table 1.

There was neither operative nor 30-day postoperative mortality. The difference of the mean operative time in both groups was statistically insignificant (*P* ≥ 0.05). The number of readmissions in delayed treatment group B was three times in 6 patients (10%), twice in 14 patients (23.3%), and once in 40 patients (66.7%) while being once only in patients in early treatment group A. Therefore, the mean total hospital stays in days for patients in group B were 5.7 ± 2.32 days compared with 2.4 ± 1.1 days in group A with significant distribution (*P* ≤ 0.0001).

The complications were traced as operative and postoperative (Table 2). Bleeding, conversion to open technique, and spillage of stones were the operative complications encountered in this study with neither major gut nor vessel injuries. The postoperative complications in our study were biliary leakage and port-site wound infections. In group A with early laparoscopic cholecystectomy, the overall complication rate was a little bit higher than that in group B with delayed laparoscopic cholecystectomy, yet the distribution was still insignificant (*P* = 0.068).

The overall patient's satisfaction was 92.66 ± 6.8 in group A compared with 75.34 ± 12.85 in group B and this distribution was significant (*P* ≤ 0.0001). As shown in Table 3, the authors observed that the recurrent attacks biliary pain and the hospital readmission let only 66.7% of patients in group B be satisfied compared with 98% of patients in group A. In case of length of hospital stay, 70% of patients in group B were satisfied compared with 95% of patients in group A while, due to operative outcome, it was observed that 90% of patients in group B were satisfied compared with 85% of patients in group A.

## 5. Discussion

Acute cholecystitis is a common cause of abdominal pain and unless treated promptly, patients may develop complications such as gangrenous, perforated, or emphysematous cholecystitis. Because of the increased morbidity and mortality of complicated cholecystitis, early diagnosis and treatment are essential for optimal patient care [5].

In the present study, the authors relied on three limbs of evaluation to categorize their patients with acute cholecystitis [2]. Clinical data included fever and pain in the right upper quadrant together with tenderness and may be accompanied by palpable mass according to the severity of the disease. Laboratory tests were C-reactive protein (CRP) and total leucocytic count (TLC). Ultrasound finding of acute cholecystitis in our patients was enlarged gallbladder with long axis measured 9 cm and wall thickness measured 8 mm with trilaminar characteristic. The presence of pericystic fluid indicated more advanced disease. These parameters of grading were in agreement with other studies of the same interest [2, 6, 7].

Surgical interference in early laparoscopic cholecystectomy patients was performed within 72 hours of occurrence of symptoms in the present study that came in concordance with other studies [4, 8, 9]. Early laparoscopic cholecystectomy within 5 days of onset of symptoms in acute phase has proved superior to open cholecystectomy [10] and early laparoscopic cholecystectomy within 4 days of onset of symptoms has been shown to reduce a number of complications and conversion rate while laparoscopic cholecystectomy performed within 24 hours resulted in more satisfactory outcome [11].

According to the updated Tokyo Guidelines 2013 (TG13), early laparoscopic cholecystectomy is indicated for patients with grade I mild acute cholecystitis because laparoscopic cholecystectomy can be performed in most of these patients. Early laparoscopic cholecystectomy within 72 h after the onset of acute cholecystitis is required in patients with grade II moderate acute cholecystitis in experienced centers [1, 12]. Accordingly, our policy for managing patients in the present study came in agreement with Tokyo Guidelines 2013 (TG13) for grades I and II acute cholecystitis.

Complications of laparoscopic cholecystectomy include early and late complications [13]. Early complications include complications due to port entry, bowel injuries, and bleeding and biliary complications include spilled gallstones, biliary leaks, and bile duct injuries. The complications can be minimized with careful patient selection, meticulous operative dissection, and judicious use of cholangiography along with sound surgical judgment [13, 14]. We observed that operative bleeding, conversion to open technique, and spillage of stones due to gallbladder perforation were the operative complications encountered in this study with similar incidence rates as previously reported [13, 15, 16]. Studies of the same interest reported that these operative and other major complications are much more encountered with advancing disease pathology as in grade II and more [1, 2, 6, 12, 15, 16].

It was stated that delayed complications or postoperative complications included port-site or wound infection, biliary leak, intra-abdominal collection, ileus, chest infection, postcholecystectomy syndrome, and CBD stricture [13, 14]. Neither common bile duct injury nor intra-abdominal collection was observed in our patients; however, wound infection and biliary leakage were observed in group A more than group B but without significant distribution and with incidence rate comparable with other reported data [13–16].

An interesting work performed at King Hussein Medical Center, Jordan, comparing the early and delayed approaches in management of acute cholecystitis stated that the early approach had the advantage of offering the patients a definitive treatment during the index admission while reducing the overall total hospital stay and avoiding the problems of failure of delayed therapy. This may translate into an economic benefit and better patient satisfaction when compared with delayed therapy [17].

Regarding the operative and postoperative complications in the study groups, the difference concerning patient's satisfaction for surgical outcome was statistically insignificant. We observed that the mean total hospital stays as a result of repeated readmission and recurrent attacks biliary pain were higher for patients in group B than in group A with significant distribution. Therefore, the overall patient's satisfaction regarding surgical outcome, recurrent attacks biliary pain, repeated readmission, and the length of hospital stay was in favor of patients with early surgical intervention [18]. Our data concerning patient's preference and satisfaction came in concordance with other published results of the same interest [17–20].

## 6. Conclusion

Early laparoscopic cholecystectomy within 72 hours of onset of symptoms has both surgical and patient's preference advantages and should be the preferred approach for patients managed by surgeons with adequate experience. Early laparoscopic cholecystectomy resulted in significant reduction in length of hospital stay and accepted rate of operative complications and conversion rates when compared with delayed techniques. The overall patient's satisfaction regarding surgical outcome, recurrent attacks biliary pain, repeated readmission, and the length of hospital stay is in favor of patients with early surgical intervention.

## Figures and Tables

**Table 1:** Grades and severity of acute cholecystitis in both groups A and B.

Item	Group A		Group B	
	Male	Female	Male	Female
Grade I	7	24	10	20
Grade II	8	21	8	22

**Table 2:** Operative and postoperative complications in both groups.

Complication	Group A (early)		Group B (delayed)		*P* value	*T* value
	Number	%	Number	%		
Bleeding	6	(10)	4	(6.7)	**0.068** **(NS)**	**2.1**
Conversion	3	(5)	1	(1.7)		
Stone spillage	4	(6.7)	2	(3.4)		
Leak	3	(5)	2	(3.4)		
Infection	3	(5)	2	(3.4)		

**Table 3:** Patient's satisfaction.

Item	Group A	Group B	*P* value	*T* value
Pain	98	66	0.0001 (S)	9.22
Readmission	95	70		
Operative outcome	85	90		
Mean	92.66	75.34		
SD	±6.8	±12.85		

## References

[1] Yamashita Y, Takada T, Strasberg SM (2013). TG13 surgical management of acute cholecystitis. *Journal of Hepato-Biliary-Pancreatic Sciences*.

[2] Hirota M, Takada T, Kawarada Y (2007). Diagnostic criteria and severity assessment of acute cholecystitis: Tokyo guidelines. *Journal of Hepato-Biliary-Pancreatic Surgery*.

[3] Yokoe M, Takada T, Strasberg SM (2012). New diagnostic criteria and severity assessment of acute cholecystitis in revised Tokyo guidelines. *Journal of Hepato-Biliary-Pancreatic Sciences*.

[4] Gurusamy K, Samraj K, Gluud C, Wilson E, Davidson BR (2010). Meta-analysis of randomized controlled trials on the safety and effectiveness of early versus delayed laparoscopic cholecystectomy for acute cholecystitis. *British Journal of Surgery*.

[5] Charalel RA, Jeffrey RB, Shin LK (2011). Complicated cholecystitis: the complementary roles of sonography and computed tomography. *Ultrasound Quarterly*.

[6] Gorka R, Azad T (2013). Factors influencing complications and conversion rates following laparoscopic cholecystectomy in acute cholecystitis. *East and Central African Journal of Surgery*.

[7] Yao Z, Hu K, Huang P (2014). Delayed laparoscopic cholecystectomy is safe and effective for acute severe calculous cholecystitis in patientswith advanced cirrhosis a single center experience. *Gastroenterology Research and Practice*.

[8] Ohta M, Iwashita Y, Yada K (2012). Operative timing of laparoscopic cholecystectomy for acute cholecystitis in a Japanese institute. *Journal of the Society of Laparoendoscopic Surgeons*.

[9] Lau H, Lo CY, Patil NG, Yuen WK (2006). Early versus delayed-interval laparoscopic cholecystectomy for acute cholecystitis: a metaanalysis. *Surgical Endoscopy and Other Interventional Techniques*.

[10] Laporte S, Navarro F (2002). What is the best timing to perform laparoscopic cholecystectomy in acute cholecystitis?. *Journal de chirurgie*.

[11] Soomro AH, Memon AA, Malik KA, Devi B (2005). Role of laparoscopic cholecystectomy in the management of acute cholecystitis. *Journal of Liaquat University of Medical and Health Sciences*.

[12] Yokoe M, Takada T, Strasberg SM (2013). TG13 diagnostic criteria and severity grading of acute cholecystitis (with videos). *Journal of Hepato-Biliary-Pancreatic Sciences*.

[13] Memon AA, Maheshwari T, Lal K, Memon ZY, Tariq A (2013). Complications of laparoscopic cholecystectomy in acute cholecystitis. *Medical Channel*.

[14] Hinduja TK, Shaikh NA, Shaikh SM, Soomro I, Jalbani MH (2008). Early laparoscopic cholecystectomy. *The Professional Medical Journal*.

[15] Shamim M, Memon AS, Dahri MM (2006). Complications of laparoscopic cholecystectomy. *Pakistan Journal Of Surgery*.

[16] Mohammad S, Hinduja T, Fatima S (2008). Complications of laparoscopic Cholecystectomy in acute cholecystitis. *Journal of Surgery Pakistan*.

[17] Al-Faouri AF, Halasa SA, Al-Hourani SA, Al-Mnaizel TS (2012). Early versus delayed laparoscopic cholecystectomy for management of acute calculus cholecystitis our experience at King Hussein Medical Center. *Journal of the Royal Naval Medical Service*.

[18] Calland JF, Tanaka K, Foley E (2001). Outpatient laparoscopic cholecystectomy: patient outcomes after implementation of a clinical pathway. *Annals of Surgery*.

[19] Durántez FD, García MA, Cuéllar AN, Robles JM, Graua JMS, Ruiz FJP (2013). Day surgery laparoscopic cholecystectomy Comparative analysis in two consecutive periods in a cohort of 1132 patients. *Ambulatory Surgery*.

[20] Sharma A, Hayden JD, Reese RA, Sedman PC, Royston CMS, O'Boyle CJ (2004). Prospective comparison of ambulatory with inpatient laparoscopic cholecystectomy: outcome, patient preference and satisfaction. *Ambulatory Surgery*.

